# The genome sequence of the house spider, *Eratigena atrica* (C.L.Koch, 1843) (Araneae: Agelenidae)

**DOI:** 10.12688/wellcomeopenres.24856.1

**Published:** 2025-09-10

**Authors:** Geoff S. Oxford, Natural History Museum Genome Acquisition Lab, Wellcome Sanger Institute Tree of Life Management, Samples and Laboratory team, Wellcome Sanger Institute Scientific Operations: Sequencing Operations, Wellcome Sanger Institute Tree of Life Core Informatics team, Tree of Life Core Informatics collective, Darwin Tree of Life Consortium

**Affiliations:** 1 Department of Biology, University of York, York, England, UK

**Keywords:** Eratigena atrica; house spider; genome sequence; chromosomal; Araneae

## Abstract

We present a genome assembly from a male specimen of *Eratigena atrica* (house spider; Arthropoda; Arachnida; Araneae; Agelenidae). The genome sequence has a total length of 4 947.42 megabases. Most of the assembly (98.73%) is scaffolded into 22 chromosomal pseudomolecules, including the X _1_ and X _2_ sex chromosomes. The mitochondrial genome was also assembled, with a length of 14.25 kilobases.

## Species taxonomy

Eukaryota; Opisthokonta; Metazoa; Eumetazoa; Bilateria; Protostomia; Ecdysozoa; Panarthropoda; Arthropoda; Chelicerata; Arachnida; Araneae; Araneomorphae; Entelegynae; RTA clade; Agelenidae; *Eratigena*; *Eratigena atrica* (C.L.Koch, 1843) (NCBI:txid1698855)

## Background

**Figure 1.**
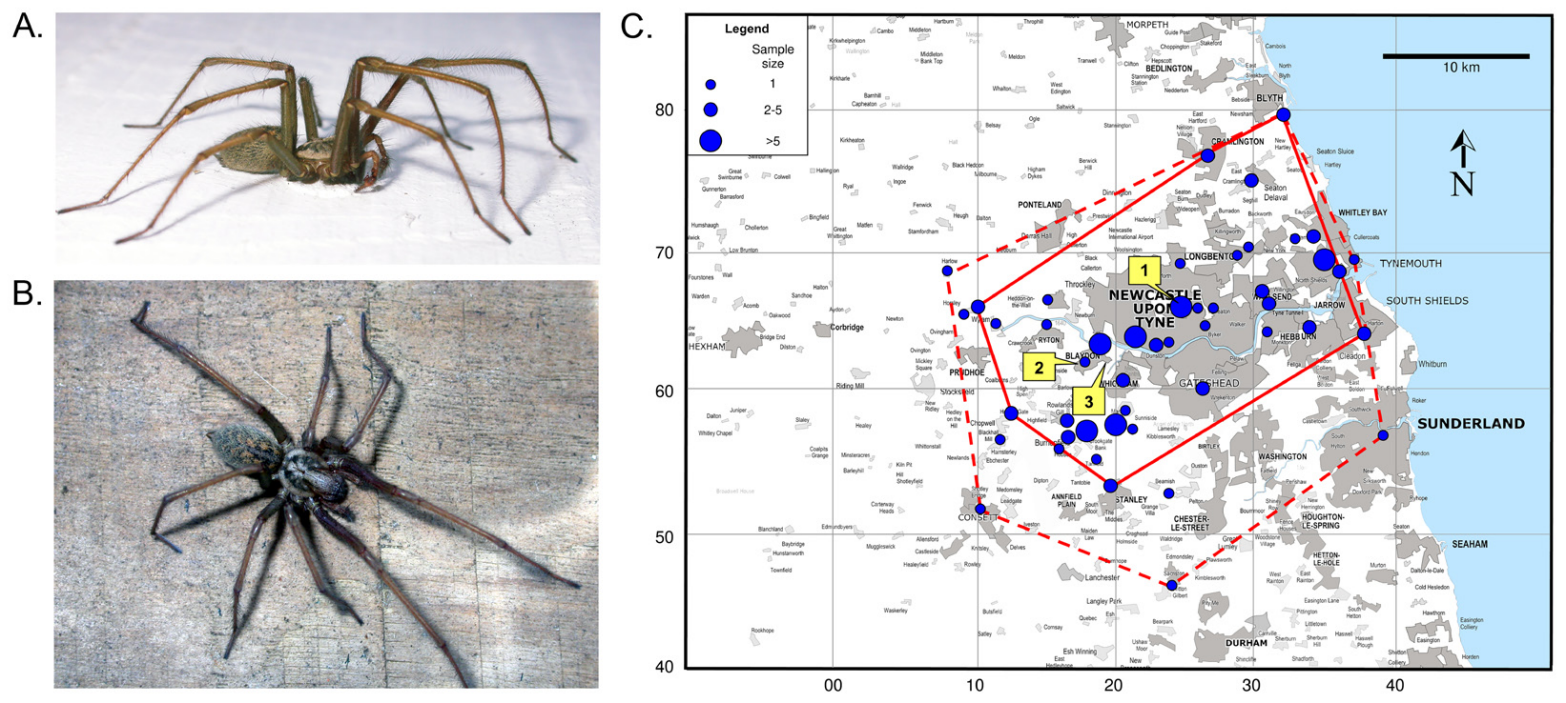
**A.** *Eratigena atrica* (male) showing the relatively long, slender legs. **B.** *E. atrica* (male) illustrating the general pattern and coloration of cephalothorax and abdomen. **C.** Distribution of *E. atrica* in the area around Newcastle-upon-Tyne, Northern England. Sample size is reflected by symbol size. The dashed-line polygon indicates the known range of the species, whereas the solid-line polygon shows the core range (see Oxford & Smith (2014) for more details). Marginal numbers are hectad grid squares within 100 km square NZ of the National Grid. This figure is reproduced, with permission, from Oxford & Smith (2014).

*Eratigena atrica* is a large, brown and hairy house spider whose apparently sudden appearance during late summer and autumn many homeowners dread. Together with *E. duellica* and *E. saeva*, it forms the *Eratigena atrica* group of closely related species ( Merrett, 1980). All three are identical in their overall appearance and life cycles but can be distinguished morphologically using palp and epigyne features ( Oxford, 2023). They are amongst Britain’s largest spiders, with body lengths of 10–14 mm in mature males and 11–16 mm in mature females. Mature females have a leg length of approximately twice their body length, whereas in males, legs are roughly three times their body length ( Bee *et al.*, 2020, see Figure 1 A). The overall appearance is brownish/grey, but the abdomen has paler patches that, towards the rear, become anterior-facing chevrons ( Bee *et al.*, 2020, see Figure 1 B). Egg sacs are laid in spring and spiders of both sexes overwinter as half-grown juveniles. They mature the following August/September, males slightly ahead of females. Mating occurs in the female’s web, where the male may cohabit for a while. Males then die and females overwinter with stored sperm. With the arrival of spring, they commence laying a series of egg sacs, each containing some 60 to 80 eggs. It is possible that some females survive for a third winter. Males, therefore, live for about 18 months and females typically two years or more ( Oxford, 2011). Although large house spiders are mostly associated in the public’s eye with human habitation and environs, they also thrive in many other habitats far from immediate human influences, for example under stones and logs, in tree cavities and beneath overhanging earth banks.

Bolzern *et al*. (2013) proposed on both morphological and molecular grounds that these species should be moved from their previous genus ( *Tegenaria*) to a new one, *Eratigena*, and controversially, that the three species in the *E. atrica* group belonged to a single, albeit variable, species, *E. atrica*. The latter suggestion was later refuted ( Oxford & Bolzern, 2018), and the World Spider Catalog once again recognises three separate species. The causes of the confusion were: (a), morphological character differences need to be viewed from exactly the right angle for a secure identification ( Oxford, 2023), and (b), *E. saeva* and *E. duellica* have hybridised during their evolutionary history and do so currently where their distributions overlap, leading to a partial sharing of mtDNA (barcoding) haplotypes ( Croucher *et al.*, 2004; Oxford & Bolzern, 2018). However, there is no evidence of *E. atrica* hybridising with the other two species in the group.

Globally, *E. atrica* is widespread in continental Europe ( Araneae – Spiders of Europe) and occurs in the Republic of Ireland, where it was probably introduced centuries ago ( Oxford, 2011; Oxford & Chesney, 1994). It was also introduced to, and has become established in, North America ( World Spider Catalog). In Britain, the most common and widespread species are *E. saeva* and *E. duellica* and, for many years, *E. atrica* was considered an occasional import. It was typically reported from post offices and garden centres, with no evidence of viable populations establishing. This remained the case until Smith & Oxford (2009) discovered a self-sustaining population in Burnopfield, County Durham. This turned out to be on the periphery of a much larger population covering some 700 km ^2^ (with a core of approximately 400 km ^2^) centred on Newcastle upon Tyne ( Oxford & Smith, 2014) ( Figure 1 C). A second population has been identified in the Perth/Dundee region of eastern Scotland but is not yet fully mapped. Both populations are almost certainly results of accidental importation from Ireland or continental Europe in historical times as both Newcastle upon Tyne and Dundee have long been important ports ( Oxford & Smith, 2014).

The genome of *Eratigena atrica* was sequenced as part of the Darwin Tree of Life Project, a collaborative effort to sequence all named eukaryotic species in the Atlantic Archipelago of Britain and Ireland. Here we present a chromosomally complete genome sequence for *Eratigena atrica* based on one male specimen from Scone, Perth, Scotland. The *E. atrica* genome will provide an extremely useful benchmark against which to compare the genomes of the other two, slightly more distantly related, species in the *E. atrica* group, and will allow a fuller understanding of the barriers to reproduction between the three species.

## Methods

### Sample acquisition and DNA barcoding

The specimen used for genome sequencing was an adult male *Eratigena atrica* (specimen ID NHMUK014447011, ToLID qqEraAtri1; Figure 1), collected from Scone, Perth, Scotland, UK (latitude 56.42, longitude –3.399) on 2020-09-27. The specimen was collected by Mike Davidson (British Arachnological Society) and identified by Geoff Oxford (University of York). A second specimen was used for Hi-C sequencing (specimen ID NHMUK014447013, ToLID qqEraAtri3), and a third specimen was used for RNA sequencing (specimen ID NHMUK014447014, ToLID qqEraAtri4). Specimens qqEraAtri3 and qqEraAtri4 were collected from Marley Hill, Co. Durham, England, UK (latitude 54.616, longitude –1.573) on 2020-09-28, and Geoff Oxford collected and identified these specimens. Sample metadata were collected in line with the Darwin Tree of Life project standards described by Lawniczak *et al*. (2022).

The initial identification was verified by an additional DNA barcoding process according to the framework developed by Twyford *et al.* (2024). A small sample was dissected from the specimen and stored in ethanol, while the remaining parts were shipped on dry ice to the Wellcome Sanger Institute (WSI) (see the protocol). The tissue was lysed, the COI marker region was amplified by PCR, and amplicons were sequenced and compared to the BOLD database, confirming the species identification ( Crowley *et al.*, 2023). Following whole genome sequence generation, the relevant DNA barcode region was also used alongside the initial barcoding data for sample tracking at the WSI ( Twyford *et al.*, 2024). The standard operating procedures for Darwin Tree of Life barcoding are available on protocols.io.

### Nucleic acid extraction

Protocols for high molecular weight (HMW) DNA extraction developed at the Wellcome Sanger Institute (WSI) Tree of Life Core Laboratory are available on protocols.io ( Howard *et al.*, 2025). The qqEraAtri1 sample was weighed and triaged to determine the appropriate extraction protocol. Tissue from the cephalothorax was homogenised by powermashing using a PowerMasher II tissue disruptor. HMW DNA was extracted using the Automated MagAttract v2 protocol. DNA was sheared into an average fragment size of 12–20 kb following the Megaruptor®3 for LI PacBio protocol. Sheared DNA was purified by automated SPRI (solid-phase reversible immobilisation). The concentration of the sheared and purified DNA was assessed using a Nanodrop spectrophotometer and Qubit Fluorometer using the Qubit dsDNA High Sensitivity Assay kit. Fragment size distribution was evaluated by running the sample on the FemtoPulse system. For this sample, the final post-shearing DNA had a Qubit concentration of 11.8 ng/μL and a yield of 4 602.00 ng. The 260/280 spectrophotometric ratio was 1.79, and the 260/230 ratio was 0.45.

RNA was extracted from leg tissue of qqEraAtri4 in the Tree of Life Laboratory at the WSI using the RNA Extraction: Automated MagMax™ *mir*Vana protocol. The RNA concentration was assessed using a Nanodrop spectrophotometer and a Qubit Fluorometer using the Qubit RNA Broad-Range Assay kit. Analysis of the integrity of the RNA was done using the Agilent RNA 6000 Pico Kit and Eukaryotic Total RNA assay.

### PacBio HiFi library preparation and sequencing

Library preparation and sequencing were performed at the WSI Scientific Operations core. Libraries were prepared using the SMRTbell Prep Kit 3.0 (Pacific Biosciences, California, USA), following the manufacturer’s instructions. The kit includes reagents for end repair/A-tailing, adapter ligation, post-ligation SMRTbell bead clean-up, and nuclease treatment. Size selection and clean-up were performed using diluted AMPure PB beads (Pacific Biosciences). DNA concentration was quantified using a Qubit Fluorometer v4.0 (ThermoFisher Scientific) and the Qubit 1X dsDNA HS assay kit. Final library fragment size was assessed with the Agilent Femto Pulse Automated Pulsed Field CE Instrument (Agilent Technologies) using the gDNA 55 kb BAC analysis kit.

The sample was sequenced using the Sequel IIe system (Pacific Biosciences, California, USA). The concentration of the library loaded onto the Sequel IIe was in the range 40–135 pM. The SMRT link software, a PacBio web-based end-to-end workflow manager, was used to set-up and monitor the run, and to perform primary and secondary analysis of the data upon completion.

### Hi-C

***Sample preparation and crosslinking***

The Hi-C sample was prepared from 20–50 mg of frozen cephalothorax tissue of the qqEraAtri3 sample using the Arima-HiC v2 kit (Arima Genomics). Following the manufacturer’s instructions, tissue was fixed and DNA crosslinked using TC buffer to a final formaldehyde concentration of 2%. The tissue was homogenised using the Diagnocine Power Masher-II. Crosslinked DNA was digested with a restriction enzyme master mix, biotinylated, and ligated. Clean-up was performed with SPRISelect beads before library preparation. DNA concentration was measured with the Qubit Fluorometer (Thermo Fisher Scientific) and Qubit HS Assay Kit. The biotinylation percentage was estimated using the Arima-HiC v2 QC beads.

***Hi-C library preparation and sequencing***

Biotinylated DNA constructs were fragmented using a Covaris E220 sonicator and size selected to 400–600 bp using SPRISelect beads. DNA was enriched with Arima-HiC v2 kit Enrichment beads. End repair, A-tailing, and adapter ligation were carried out with the NEBNext Ultra II DNA Library Prep Kit (New England Biolabs), following a modified protocol where library preparation occurs while DNA remains bound to the Enrichment beads. Library amplification was performed using KAPA HiFi HotStart mix and a custom Unique Dual Index (UDI) barcode set (Integrated DNA Technologies). Depending on sample concentration and biotinylation percentage determined at the crosslinking stage, libraries were amplified with 10–16 PCR cycles. Post-PCR clean-up was performed with SPRISelect beads. Libraries were quantified using the AccuClear Ultra High Sensitivity dsDNA Standards Assay Kit (Biotium) and a FLUOstar Omega plate reader (BMG Labtech).

Prior to sequencing, libraries were normalised to 10 ng/μL. Normalised libraries were quantified again and equimolar and/or weighted 2.8 nM pools. Pool concentrations were checked using the Agilent 4200 TapeStation (Agilent) with High Sensitivity D500 reagents before sequencing. Sequencing was performed using paired-end 150 bp reads on the Illumina NovaSeq 6000.

### RNA library preparation and sequencing

Libraries were prepared using the NEBNext ^®^ Ultra™ II Directional RNA Library Prep Kit for Illumina (New England Biolabs), following the manufacturer’s instructions. Poly(A) mRNA in the total RNA solution was isolated using oligo(dT) beads, converted to cDNA, and uniquely indexed; 14 PCR cycles were performed. Libraries were size-selected to produce fragments between 100–300 bp. Libraries were quantified, normalised, pooled to a final concentration of 2.8 nM, and diluted to 150 pM for loading. Sequencing was carried out on the Illumina NovaSeq 6000 to generate 150-bp paired-end reads.

### Genome assembly

Prior to assembly of the PacBio HiFi reads, a database of *k*-mer counts ( *k* = 31) was generated from the filtered reads using FastK. GenomeScope2 ( Ranallo-Benavidez *et al.*, 2020) was used to analyse the *k*-mer frequency distributions, providing estimates of genome size, heterozygosity, and repeat content.

The HiFi reads were assembled using Hifiasm ( Cheng *et al.*, 2021) with the --primary option. The Hi-C reads ( Rao *et al.*, 2014) were mapped to the primary contigs using bwa-mem2 ( Vasimuddin *et al.*, 2019), and the contigs were scaffolded in YaHS ( Zhou *et al.*, 2023) with the --break option for handling potential misassemblies. The scaffolded assemblies were evaluated using Gfastats ( Formenti *et al.*, 2022), BUSCO ( Manni *et al.*, 2021) and MERQURY.FK ( Rhie *et al.*, 2020). The mitochondrial genome was assembled using MitoHiFi ( Uliano-Silva *et al.*, 2023), which runs MitoFinder ( Allio *et al.*, 2020) and uses these annotations to select the final mitochondrial contig and to ensure the general quality of the sequence.

### Assembly curation

The assembly was decontaminated using the Assembly Screen for Cobionts and Contaminants ( ASCC) pipeline. TreeVal was used to generate the flat files and maps for use in curation. Manual curation was conducted primarily in PretextView and HiGlass ( Kerpedjiev *et al.*, 2018). Scaffolds were visually inspected and corrected as described by Howe *et al*. (2021). Manual corrections included 9 breaks, 33 joins, and removal of 13 haplotypic duplications. The sex chromosomes X _1_ and X _2_ were identified by PacBio read coverage and named by size. The curation process is documented at https://gitlab.com/wtsi-grit/rapid-curation. PretextSnapshot was used to generate a Hi-C contact map of the final assembly.

### Assembly quality assessment

The Merqury.FK tool ( Rhie *et al.*, 2020) was run in a Singularity container ( Kurtzer *et al.*, 2017) to evaluate *k*-mer completeness and assembly quality for the primary and alternate haplotypes using the *k*-mer databases ( *k* = 31) computed prior to genome assembly. The analysis outputs included assembly QV scores and completeness statistics.

The genome was analysed using the BlobToolKit pipeline, a Nextflow implementation of the earlier Snakemake version ( Challis *et al.*, 2020). The pipeline aligns PacBio reads using minimap2 ( Li, 2018) and SAMtools ( Danecek *et al.*, 2021) to generate coverage tracks. It runs BUSCO ( Manni *et al.*, 2021) using lineages identified from NCBI Taxonomy ( Schoch *et al.*, 2020). For the three domain-level lineages, BUSCO genes are aligned to the UniProt Reference Proteomes database ( Bateman *et al.*, 2023) using DIAMOND blastp ( Buchfink *et al.*, 2021). The genome is divided into chunks based on the density of BUSCO genes from the closest taxonomic lineage, and each chunk is aligned to the UniProt Reference Proteomes database with DIAMOND blastx. Sequences without hits are chunked using seqtk and aligned to the NT database with blastn ( Altschul *et al.*, 1990). The BlobToolKit suite consolidates all outputs into a blobdir for visualisation. The BlobToolKit pipeline was developed using nf-core tooling ( Ewels *et al.*, 2020) and MultiQC ( Ewels *et al.*, 2016), with package management via Conda and Bioconda ( Grüning *et al.*, 2018), and containerisation through Docker ( Merkel, 2014) and Singularity ( Kurtzer *et al.*, 2017).

## Genome sequence report

### Sequence data

**Figure 2.**
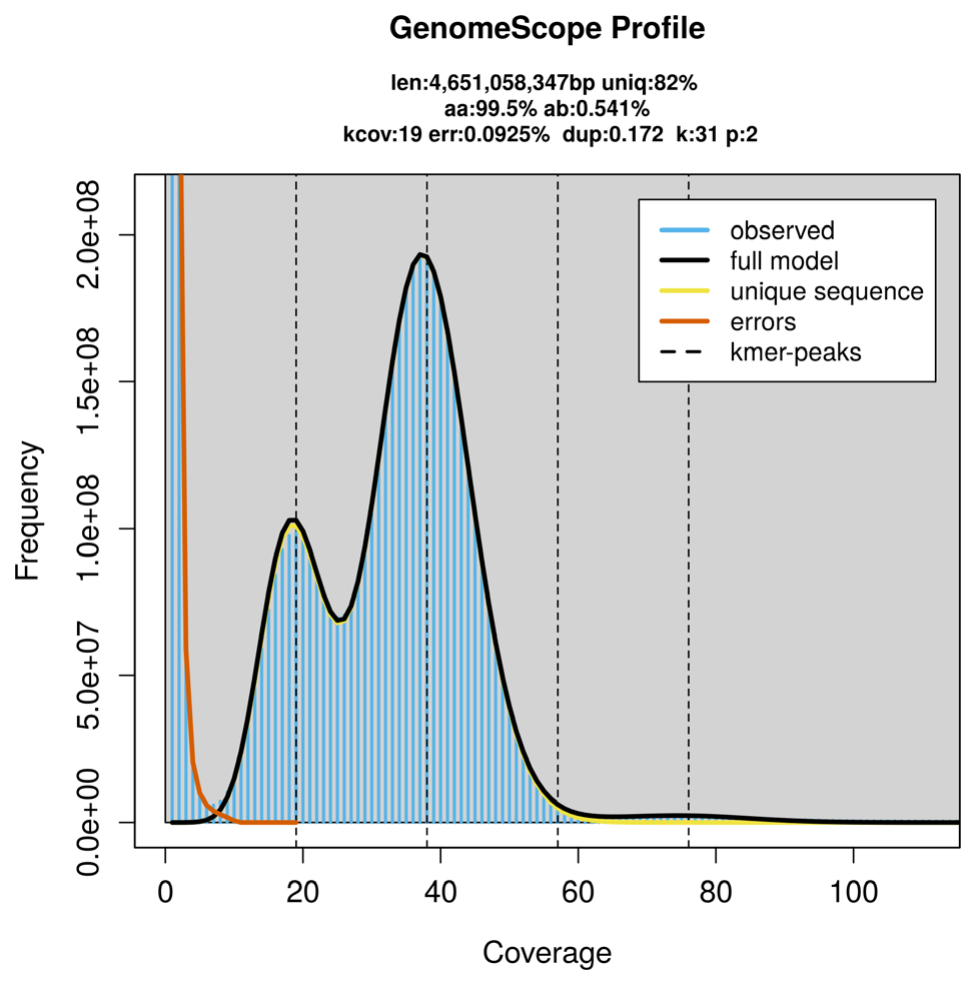
Frequency distribution of *k*-mers generated using GenomeScope2. The plot shows observed and modelled *k*-mer spectra, providing estimates of genome size, heterozygosity, and repeat content based on unassembled sequencing reads.

PacBio sequencing of the *Eratigena atrica* specimen generated 183.53 Gb (gigabases) from 20.11 million reads, which were used to assemble the genome. GenomeScope2.0 analysis estimated the haploid genome size at 4 651.06 Mb, with a heterozygosity of 0.54% and repeat content of 18.07% ( Figure 2). These estimates guided expectations for the assembly. Based on the estimated genome size, the sequencing data provided approximately 38× coverage. Hi-C sequencing produced 604.30 Gb from 4 001.97 million reads, which were used to scaffold the assembly. RNA sequencing data were also generated and are available in public sequence repositories. Table 1 summarises the specimen and sequencing details.

**Table 1.** Specimen and sequencing data for BioProject PRJEB74157.

Platform	PacBio HiFi	Hi-C	RNA-seq
**ToLID**	qqEraAtri1	qqEraAtri3	qqEraAtri4
**Specimen ID**	NHMUK014447011	NHMUK014447013	NHMUK014447014
**BioSample (source individual)**	SAMEA9066030	SAMEA9066032	SAMEA9066033
**BioSample (tissue)**	SAMEA9066105	SAMEA9066112	SAMEA9066115
**Tissue**	cephalothorax	cephalothorax	leg
**Instrument**	Sequel IIe	Illumina NovaSeq 6000	Illumina NovaSeq 6000
**Run accessions**	ERR12779270; ERR12779271; ERR12779273; ERR12779275; ERR12779272; ERR12779274	ERR12791499	ERR12791512
**Read count total**	20.11 million	4 001.97 million	71.78 million
**Base count total**	183.53 Gb	604.30 Gb	10.84 Gb

### Assembly statistics

The primary haplotype was assembled, and contigs corresponding to an alternate haplotype were also deposited in INSDC databases. The final assembly has a total length of 4 947.42 Mb in 474 scaffolds, with 621 gaps, and a scaffold N50 of 231.92 Mb ( Table 2).

**Table 2.** Genome assembly statistics.

**Assembly name**	qqEraAtri1.1
**Assembly accession**	GCA_965122215.1
**Alternate haplotype accession**	GCA_965122695.1
**Assembly level**	chromosome
**Span (Mb)**	4 947.42
**Number of chromosomes**	22
**Number of contigs**	1 095
**Contig N50**	21.42 Mb
**Number of scaffolds**	474
**Scaffold N50**	231.92 Mb
**Sex chromosomes**	X _1_ and X _2_
**Organelles**	Mitochondrion: 14.25 kb

**Figure 3.**
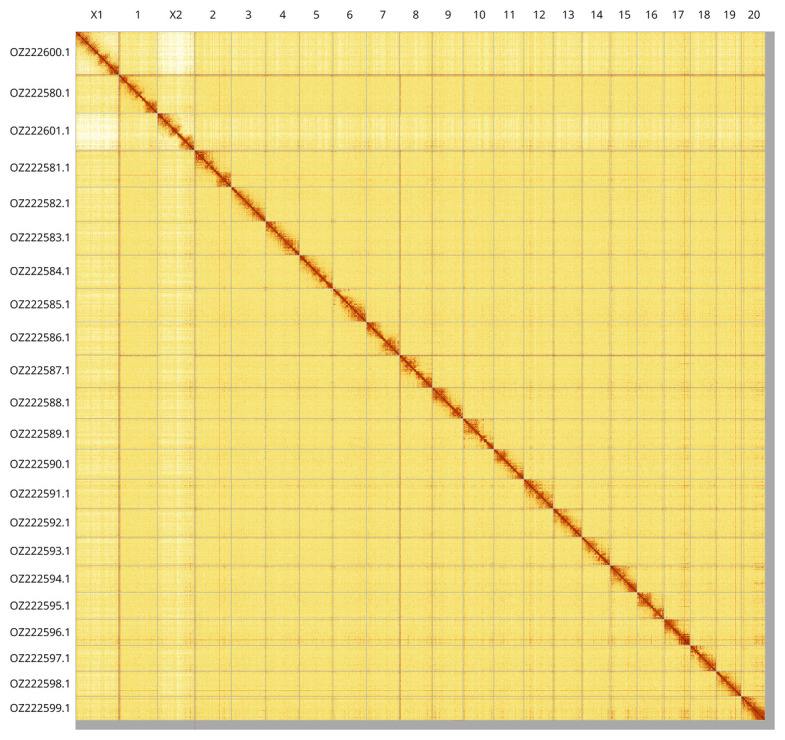
Hi-C contact map of the *Eratigena atrica* genome assembly. Assembled chromosomes are shown in order of size and labelled along the axes, with a megabase scale shown below. The plot was generated using PretextSnapshot.

Most of the assembly sequence (98.73%) was assigned to 22 chromosomal-level scaffolds, representing 20 autosomes and the X _1_ and X _2_ sex chromosomes. These chromosome-level scaffolds, confirmed by Hi-C data, are named according to size ( Figure 3; Table 3).

**Table 3.** Chromosomal pseudomolecules in the primary genome assembly of *Eratigena atrica* qqEraAtri1.

INSDC accession	Molecule	Length (Mb)	GC%
OZ222580.1	1	272.73	31
OZ222581.1	2	259.80	31.50
OZ222582.1	3	244	31.50
OZ222583.1	4	240.16	31.50
OZ222584.1	5	236.96	31.50
OZ222585.1	6	235.49	31.50
OZ222586.1	7	233.81	31.50
OZ222587.1	8	231.92	31.50
OZ222588.1	9	224.14	32
OZ222589.1	10	217.55	31.50
OZ222590.1	11	212.33	31.50
OZ222591.1	12	207.28	32
OZ222592.1	13	202.42	31.50
OZ222593.1	14	199.94	31.50
OZ222594.1	15	191.96	32.50
OZ222595.1	16	190.52	32
OZ222596.1	17	184.95	33
OZ222597.1	18	183.22	32
OZ222598.1	19	177.96	31.50
OZ222599.1	20	168.24	33.50
OZ222600.1	X _1_	306.02	32
OZ222601.1	X _2_	263.33	32

The mitochondrial genome was also assembled. This sequence is included as a contig in the multifasta file of the genome submission and as a standalone record.

**Figure 4.**
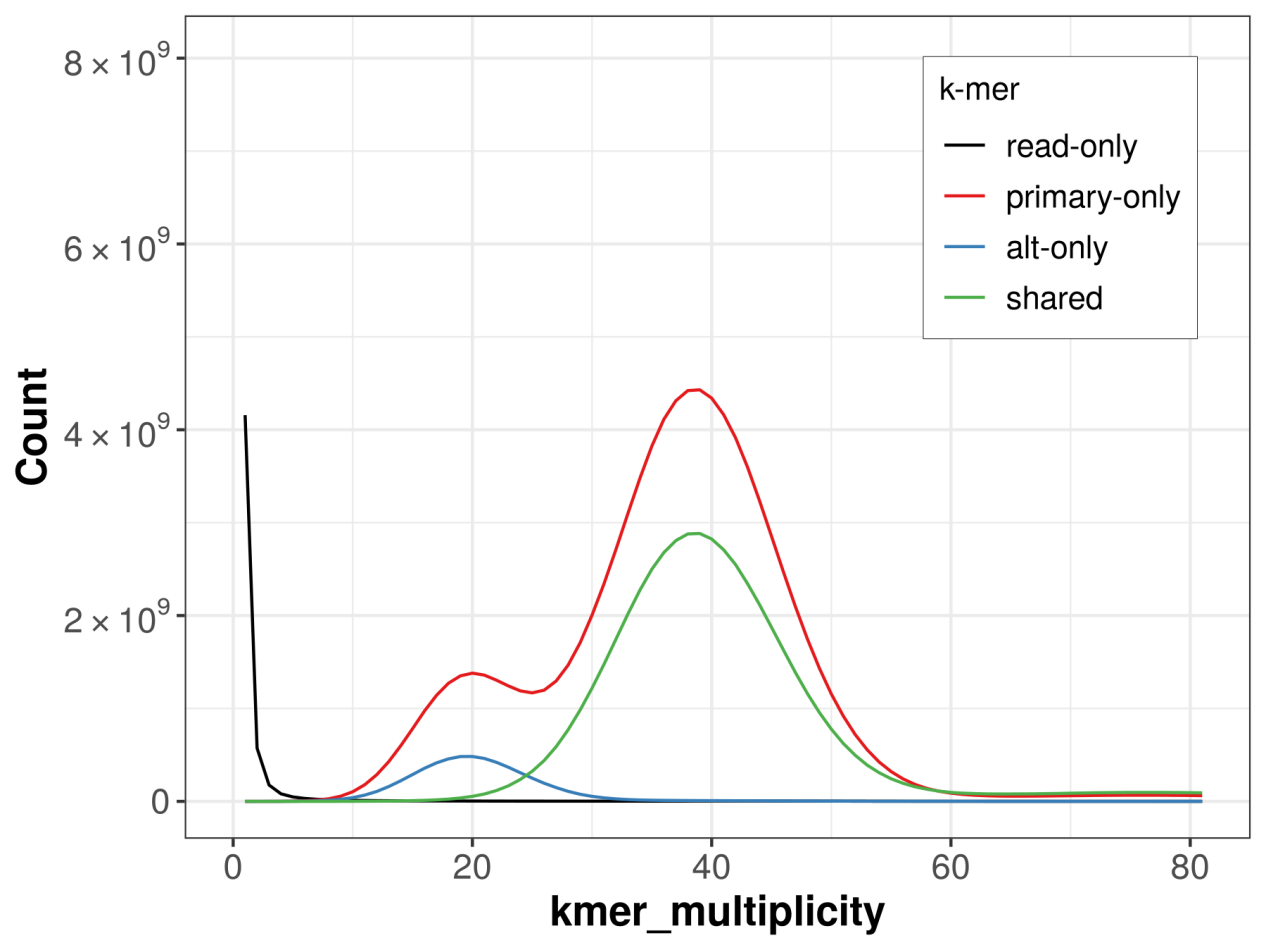
Evaluation of *k*-mer completeness using MerquryFK. This plot illustrates the recovery of *k*‐mers from the original read data in the final assemblies. The horizontal axis represents *k*‐mer multiplicity, and the vertical axis shows the number of *k*‐mers. The black curve represents *k*‐mers that appear in the reads but are not assembled. The green curve corresponds to *k*‐mers shared by both haplotypes, and the red and blue curves show *k*‐mers found only in one of the haplotypes.

The combined primary and alternate assemblies achieve an estimated QV of 67.0. The *k*-mer completeness is 93.06% for the primary assembly, 37.35% for the alternate haplotype, and 99.70% for the combined assemblies ( Figure 4).

**Figure 5.**
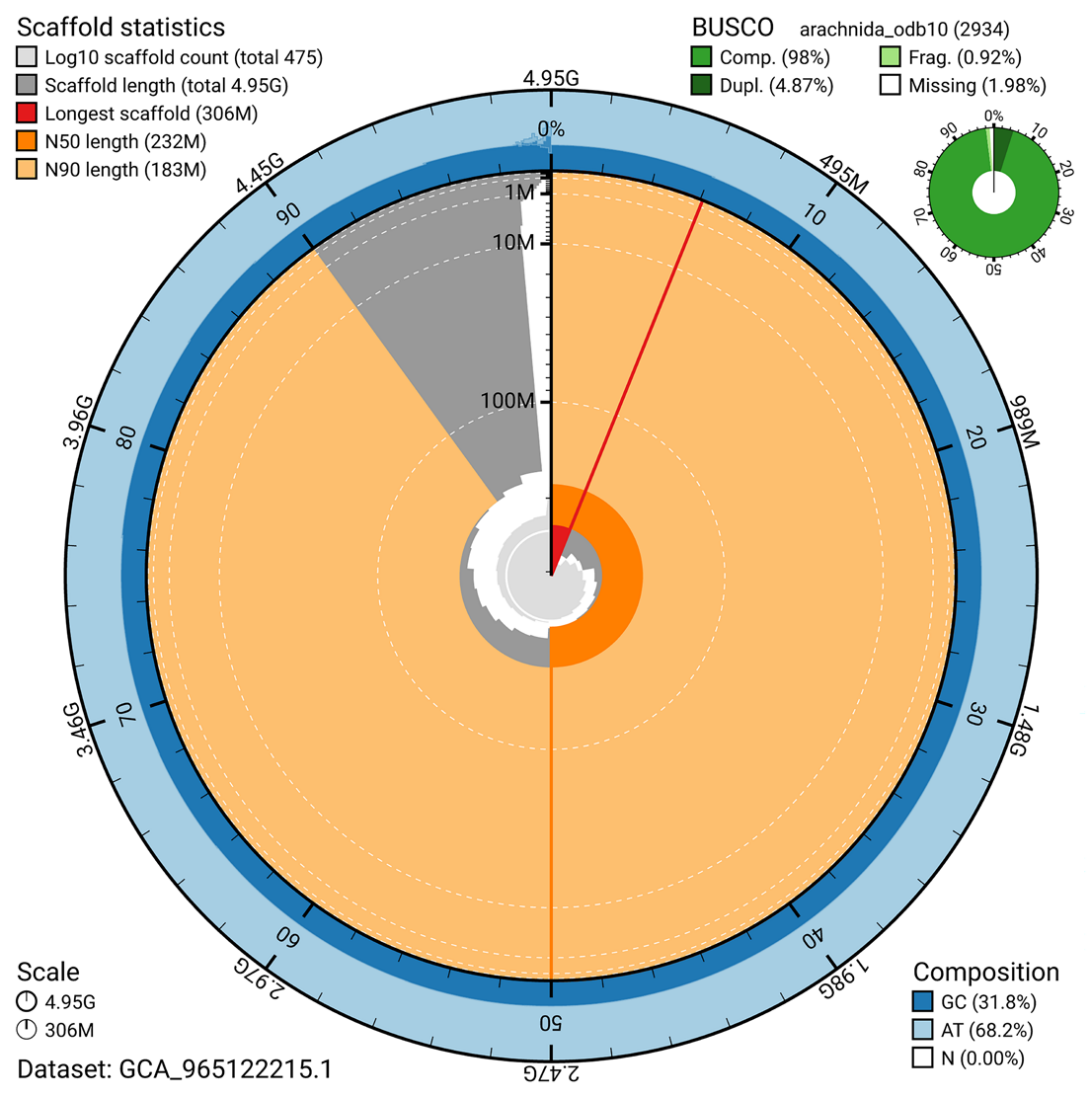
Assembly metrics for qqEraAtri1.1. The BlobToolKit snail plot provides an overview of assembly metrics and BUSCO gene completeness. The circumference represents the length of the whole genome sequence, and the main plot is divided into 1 000 bins around the circumference. The outermost blue tracks display the distribution of GC, AT, and N percentages across the bins. Scaffolds are arranged clockwise from longest to shortest and are depicted in dark grey. The longest scaffold is indicated by the red arc, and the deeper orange and pale orange arcs represent the N50 and N90 lengths. A light grey spiral at the centre shows the cumulative scaffold count on a logarithmic scale. A summary of complete, fragmented, duplicated, and missing BUSCO genes in the arachnida_odb10 set is presented at the top right. An interactive version of this figure can be accessed on the BlobToolKit viewer.

**Figure 6.**
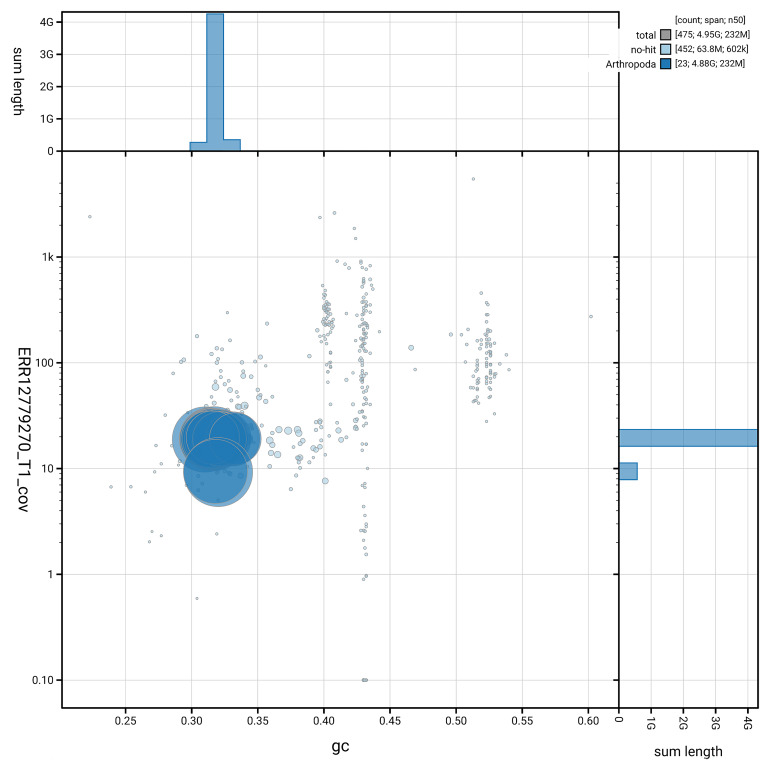
BlobToolKit GC-coverage plot for qqEraAtri1.1. Blob plot showing sequence coverage (vertical axis) and GC content (horizontal axis). The circles represent scaffolds, with the size proportional to scaffold length and the colour representing phylum membership. The histograms along the axes display the total length of sequences distributed across different levels of coverage and GC content. An interactive version of this figure is available on the BlobToolKit viewer.

BUSCO v.5.5.0 analysis using the arachnida_odb10 reference set ( *n* = 2 934) identified 98.0% of the expected gene set (single = 93.1%, duplicated = 4.9%). The snail plot in Figure 5 summarises the scaffold length distribution and other assembly statistics for the primary assembly. The blob plot in Figure 6 shows the distribution of scaffolds by GC proportion and coverage.

Table 4 lists the assembly metric benchmarks adapted from Rhie *et al.* (2021) the Earth BioGenome Project Report on Assembly Standards September 2024. The EBP metric, calculated for the primary assembly, is **7.C.Q67**, meeting the recommended reference standard.

**Table 4.** Earth Biogenome Project summary metrics for the *Eratigena atrica* assembly.

Measure	Value	Benchmark
EBP summary (primary)	7.C.Q67	6.C.Q40
Contig N50 length	21.42 Mb	≥ 1 Mb
Scaffold N50 length	231.92 Mb	= chromosome N50
Consensus quality (QV)	Primary: 67.0; alternate: 65.7; combined: 67.0	≥ 40
*k*-mer completeness	Primary: 93.06%; alternate: 37.35%; combined: 99.70%	≥ 95%
BUSCO	C:98.0% [S:93.1%; D:4.9%]; F:0.9%; M:1.1%; n:2 934	S > 90%; D < 5%
Percentage of assembly assigned to chromosomes	98.73%	≥ 90%

### Wellcome Sanger Institute – Legal and Governance

The materials that have contributed to this genome note have been supplied by a Darwin Tree of Life Partner. The submission of materials by a Darwin Tree of Life Partner is subject to the **‘Darwin Tree of Life Project Sampling Code of Practice’**, which can be found in full on the Darwin Tree of Life website. By agreeing with and signing up to the Sampling Code of Practice, the Darwin Tree of Life Partner agrees they will meet the legal and ethical requirements and standards set out within this document in respect of all samples acquired for, and supplied to, the Darwin Tree of Life Project. Further, the Wellcome Sanger Institute employs a process whereby due diligence is carried out proportionate to the nature of the materials themselves, and the circumstances under which they have been/are to be collected and provided for use. The purpose of this is to address and mitigate any potential legal and/or ethical implications of receipt and use of the materials as part of the research project, and to ensure that in doing so we align with best practice wherever possible. The overarching areas of consideration are:

- Ethical review of provenance and sourcing of the material
- Legality of collection, transfer and use (national and international)

Each transfer of samples is further undertaken according to a Research Collaboration Agreement or Material Transfer Agreement entered into by the Darwin Tree of Life Partner, Genome Research Limited (operating as the Wellcome Sanger Institute), and in some circumstances, other Darwin Tree of Life collaborators.

## Data Availability

European Nucleotide Archive: Eratigena atrica. Accession number PRJEB74157. The genome sequence is released openly for reuse. The *Eratigena atrica* genome sequencing initiative is part of the Darwin Tree of Life Project (PRJEB40665) and the Sanger Institute Tree of Life Programme (PRJEB43745). All raw sequence data and the assembly have been deposited in INSDC databases. The genome will be annotated using available RNA-Seq data and presented through the Ensembl pipeline at the European Bioinformatics Institute. Raw data and assembly accession identifiers are reported in Table 1 and Table 2. Production code used in genome assembly at the WSI Tree of Life is available at https://github.com/sanger-tol. Table 5 lists software versions used in this study.

## Author information

Contributors are listed at the following links:

- Members of the Natural History Museum Genome Acquisition Lab
- Members of the Darwin Tree of Life Barcoding collective
- Members of the Wellcome Sanger Institute Tree of Life Management, Samples and Laboratory team
- Members of Wellcome Sanger Institute Scientific Operations – Sequencing Operations
- Members of the Wellcome Sanger Institute Tree of Life Core Informatics team
- Members of the Tree of Life Core Informatics collective
- Members of the Darwin Tree of Life Consortium
