# Supplementary material for: The genome sequence of the house spider, Eratigena atrica (C.L.Koch, 1843) (Araneae: Agelenidae)
Source: Wellcome Open Res. 2025 Sep 10;10:503. [Version 1] doi: 10.12688/wellcomeopenres.24856.1 (PMC12553971; doi:10.12688/wellcomeopenres.24856.1)
Supplement: Peer review history

# Reviewer report: Reviewer response for version 1

**DOI:** 10.21956/wellcomeopenres.27380.r132974

**Published:** 2025-10-25

**Authors:** Guilherme Gainett

**Affiliations:** 1 Harvard Medical School, Boston, Massachusetts, USA

Background: 

 -hairy is inadequate term for the setae of spiders.

 -"All three are similar in their..."

 Report

 -Bloon plot: There is a large scaffold that in assigned as no-hit instead of Arthropoda. Could you please clarify this point?

 The data is well presented, and the methods are also well documented, including tutorials. Raw data is available at ENA Browser. It wasn't clear to me what the RNA data was used for. Is it for the upcoming annotation? Could the annotation be provided in this publication? It would be helpful.

 No further concerns.

Are sufficient details of methods and materials provided to allow replication by others?

Yes

Is the rationale for creating the dataset(s) clearly described?

Yes

Are the datasets clearly presented in a useable and accessible format?

Yes

Are the protocols appropriate and is the work technically sound?

Yes

Reviewer Expertise:

Evolution, development, genomics, sensory biology

I confirm that I have read this submission and believe that I have an appropriate level of expertise to confirm that it is of an acceptable scientific standard.

# Reviewer report: Reviewer response for version 1

**DOI:** 10.21956/wellcomeopenres.27380.r132972

**Published:** 2025-09-24

**Authors:** Marc Domènech

**Affiliations:** 1 Universitat de Barcelona, Barcelona, Spain

The article presents the chromosome-level genome of the spider Eratigena atrica. The genome is arranged in 22 pseudochromosomes. This species is part of a species complex, and the status and relationships among the species in this complex have changed through time. This new genome will allow for a better understanding of this problem in the future.

 The introduction about the organism is complete and accurate, and does a good job at succintly explaining the taxonomic situation of the species.

 My knowledge about genomic techniques is not enough to judge the genomic part of the methods section.

Are sufficient details of methods and materials provided to allow replication by others?

Yes

Is the rationale for creating the dataset(s) clearly described?

Yes

Are the datasets clearly presented in a useable and accessible format?

Yes

Are the protocols appropriate and is the work technically sound?

Yes

Reviewer Expertise:

Arachnology, Taxonomy

I confirm that I have read this submission and believe that I have an appropriate level of expertise to confirm that it is of an acceptable scientific standard.
